# Technical Note: spektr 3.0—A computational tool for x-ray spectrum modeling and analysis

**DOI:** 10.1118/1.4955438

**Published:** 2016-07-21

**Authors:** J. Punnoose, J. Xu, A. Sisniega, W. Zbijewski, J. H. Siewerdsen

**Affiliations:** Department of Biomedical Engineering, Johns Hopkins University, Baltimore, Maryland 21205

**Keywords:** x-ray spectrum, spectral modeling, radiation dose, spektr, TASMIP, TASMICS

## Abstract

**Purpose:**

A computational toolkit (spektr 3.0) has been developed to calculate x-ray spectra based on the tungsten anode spectral model using interpolating cubic splines (TASMICS) algorithm, updating previous work based on the tungsten anode spectral model using interpolating polynomials (TASMIP) spectral model. The toolkit includes a matlab (The Mathworks, Natick, MA) function library and improved user interface (UI) along with an optimization algorithm to match calculated beam quality with measurements.

**Methods:**

The spektr code generates x-ray spectra (photons/mm^2^/mAs at 100 cm from the source) using TASMICS as default (with TASMIP as an option) in 1 keV energy bins over beam energies 20–150 kV, extensible to 640 kV using the TASMICS spectra. An optimization tool was implemented to compute the added filtration (Al and W) that provides a best match between calculated and measured x-ray tube output (mGy/mAs or mR/mAs) for individual x-ray tubes that may differ from that assumed in TASMICS or TASMIP and to account for factors such as anode angle.

**Results:**

The median percent difference in photon counts for a TASMICS and TASMIP spectrum was 4.15% for tube potentials in the range 30–140 kV with the largest percentage difference arising in the low and high energy bins due to measurement errors in the empirically based TASMIP model and inaccurate polynomial fitting. The optimization tool reported a close agreement between measured and calculated spectra with a Pearson coefficient of 0.98.

**Conclusions:**

The computational toolkit, spektr, has been updated to version 3.0, validated against measurements and existing models, and made available as open source code. Video tutorials for the spektr function library, UI, and optimization tool are available.

## 1. INTRODUCTION

The spektr toolkit^1^ for calculation and analysis of x-ray spectra in the diagnostic energy range has been employed in a variety of imaging applications, such as modeling of imaging performance,^1–6^ analysis of spectral/dual-energy imaging,^7–17^ phase contrast imaging,^18,19^ development of novel x-ray detectors,^20–28^ modeling of x-ray scatter and beam-hardening corrections,^29–33^ development of 3D image reconstruction algorithms,^34–37^ development of new contrast agents,^38,39^ and modeling (and reduction) of radiation dose.^40–53^ At its heart, spektr 2.0 is a matlab (The Mathworks, Natick, MA) function library and user interface (UI) implementation of the tungsten anode spectral model using interpolating polynomials (TASMIP),^54^ which interpolates the measurements of Fewell *et al.*^55^ (2 keV bins) and uses polynomial fitting to approximate the photon fluence per mAs in 1 keV bins from 10 to 140 keV at x-ray tube potentials ranging from 30 to 140 kV as described in the work of Boone.

Recent work extends TASMIP to a new spectral model developed by Hernandez and Boone^56^ dubbed the tungsten anode spectral model using interpolating cubic splines (TASMICS), which uses piecewise third-order polynomial spline approximations analogous to the original TASMIP to compute the number of photons in each energy bin as a function of tube potential. Based on a Monte Carlo (MC) simulation,^57^ TASMICS avoids possible systematic measurement errors (arising, for example, from charge pile up and electronic noise) and allows higher energy resolution, extension of the x-ray tube potential to 640 kV, and generation of minimally filtered (0.8 mm Be) spectra.

In light of this improved spectral model, this Technical Note reports an update to the spektr toolkit with TASMICS as the default method for spectral calculation. The code (referred to as spektr 3.0) also includes a new optimization tool to assist with a common issue faced by TASMIP/TASMICS/spektr users: how to model and match the exposure characteristics of a particular x-ray tube that differs from that in the underlying TASMICS simulation. The code was developed and validated using matlab release 2013b. The spektr 3.0/TASMICS implementation is detailed below, and the code is freely available for download at: http://istar.jhu.edu/downloads/. Video tutorials for the spektr function library, GUI, and optimization tool are available at the same link and at the following YouTube links:

1. https://www.youtube.com/watch?v=84DJndsj9CY,
2. https://www.youtube.com/watch?v=fXenb_LNMKM,
3. https://www.youtube.com/watch?v=Kn588r4arTM.

## 2. THE spektr 3.0 TOOLKIT

### 2.A. Implementation

The x-ray fluence (photons/mm^2^/mAs at 100 cm from the source) in each energy bin was drawn from the data of Hernandez and Boone^56^ and stored in *spektrTASMICSdata.mat* for beam energies 20–150 kV. The energy-dependent attenuation coefficients in *spektrMuRhoElements.mat* and *spektrMuRhoCompounds.mat* were updated to correspond to the average energy in each energy bin for a TASMICS spectrum (1.5, 2.5 keV, etc.) using values from the NIST XCom database and a cubic interpolation of NIST attenuation coefficients for selected compounds, respectively.^1,58^ All Microsoft Excel dependencies from the original spektr release^1^ have been removed in spektr 3.0. The basic spectrum calculation is computed with the function call,

spektrSpectrum(kV, [mmAl, kV _ripple],spectral_model, normalize) (1a)

where kV is the tube potential (kV), mmAl is the added Al filtration (mm), and kV_ripple is the kV ripple (%). For backward compatibility, spectral_model can be set to “TASMIP” (default = “TASMICS”) to generate the original spektr 2.0 spectrum. The default normalize flag (=1) normalizes the calculated tube output (mGy/mAs at 100 cm from the source) to match that of a spectrum calculated by spektr 2.0 (TASMIP) at the same kV. Setting the normalize flag to 0 generates a TASMICS spectrum that is not normalized to match the spektr 2.0 tube output. For example, a function call without optional arguments (i.e., kV argument only)

spektrSpectrum(70) (1b)

generates a 70 kV spectrum using the TASMICS model, with 1.6 mm Al inherent filtration, 0% kV ripple, and normalization of mGy/mAs to match that of the TASMIP/Fewell spectrum. As described below, the choice of 1.6 mm Al filtration matches the inherent filtration of TASMIP. To generate a spectrum equivalent to Eq. (1b) using the TASMIP model, spectral_model is set to TASMIP as in

spektrSpectrum(70,[0, 0],‘TASMIP’,0) (1c)

Note that the output of *spektrSpectrum*() when spectral_model is set to ‘TASMIP’ is independent of the state of the normalize parameter. Alternatively, the function call

spektrSpectrum(70,[0, 0],‘TASMICS’,0) (1d)

computes a 70 kV TASMICS spectrum with no added filtration, 0% kV ripple, and no normalization of tube output. Although TASMICS is defined for potentials across the orthovoltage range, spektr 3.0 calculations are currently capped at 150 kV for backward compatibility. The code can be extended to 640 kV by adjusting the *spektrTASMICSdata.mat* file and modifying the functions in the spektr library to operate on spectra of length [1:640]. A glossary of the main spektr functions is given in Table I along with new functions introduced in version 3.0.

**TABLE I.** Glossary of spektr 3.0 functions.

matlab function	Description
*spektr*	Launch spektr graphical UI
airKerma = *spektrAirKerma*(q)	Calculate the mGy/mAs for spectrum q at 100 cm from the focal spot
fluencePerAirKerma = *spektrFluencePerAirKerma*(q)	Calculate the fluence per air kerma for spectrum q at 100 cm from the focal spot
X = *spektrExposure*(q)	Compute mR/mAs for spectrum q at 100 cm from the focal spot
$\bar{q_{o}}/X=$ *spektrFluencePerExposure*(q)	Compute the fluence per exposure for spectrum q at 100 cm from the focal spot
[mu, rho] = *spektrMuRhoElement*(Z)	Return μ(E) and ρ for element *Z* in units of mm^−1^ and g/cm^3^, respectively
mu_rho = *spektrMuRhoCompound*([elements])	Compute μ/ρ(E) in cm^2^/g for the compound defined by the constituents in [elements]
[mu, rho] = *spektrMuRhoCompound*(compoundNumber)	Compute μ(E) and ρ in mm^−1^ and g/cm^3^, respectively, for the compound defined by the index (1–20) compoundNumber
qFiltered = *spetkrBeersCompoundsNIST*(q, [compoundFilters])	Filter spectrum q using compounds and thicknesses in [compoundFilters]
[filtered spectrum] = *spektrBeers*(q, [filters])	Filter spectrum q by the materials and thicknesses in [filters]. Outputs the filtered spectrum
[spectrum] = *spektrSpectrum*(kV p, [mmAl ripple], spectralModel, normalize)	Generate an x-ray spectrum
[mmAl, mmW] = *spektrTuner*(kV p, mAs, measurement,SDD, [filters], measurementFlag,…[estimateInherentFilters])	Compute the filtration which provides best match of calculated and measured x-ray tube output

### 2.B. Validation

**FIG. 1.**
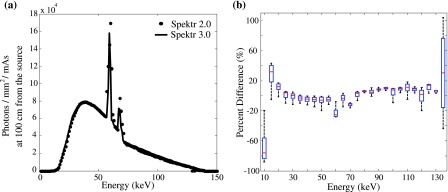
Comparison between spektr 2.0 and 3.0 implementations of TASMIP and TASMICS, respectively. (a) Spectra computed at 140 kV emphasizing the difference at tungsten *K*-edge energies. (b) Boxplot of the percent difference in photon fluence computed in each energy bin for beam energies ranging from 30 to 140 kV.

Spectra computed using spektr 3.0 (TASMICS) were compared to spektr 2.0 (TASMIP) calculations. As shown in Fig. 1(a), spectra computed with normalize set to 1 are in close agreement. Similar agreement was demonstrated for spectra computed over the range 30–140 kV (not shown; Pearson’s *R*^2^ coefficient >0.93 for all cases). The level of agreement is more fully quantified in Fig. 1(b), which plots the difference in photon fluence (x-rays/mm^2^/mAs at 100 cm from the source) in each energy bin for *spektrSpectrum*(kV) computed using spektr 2.0 and 3.0, pooling calculations over 30–140 kV in 5 kV intervals. A median discrepancy of 4.15% was observed over the range 10–150 keV, slightly higher than that reported by Hernandez and Boone.^56^ The slight discrepancy between spektr 2.0 and spektr 3.0 calculations is attributed to differences in fitting within each energy bin: TASMIP (spektr 2.0) uses a polynomial to fit photon fluence within a given bin, whereas TASMICS (spektr 3.0) uses a localized, piecewise third-order fit to the photon fluence in an energy bin.

Larger variations in the low-energy bins (10–15 keV) are likely due to measurement errors (presumably due to challenges in measurement of x-ray spectra, such as electronic noise, charge pile up, and x-ray scatter) and/or the resulting polynomial coefficients on which spektr 2.0 is based. spektr 3.0 is based on the MC simulation^57^ underlying TASMICS and does not depend on such factors. Variation in the 60 keV bin is due to the increased energy resolution in spektr 3.0. Specifically, the 2 keV energy resolution Fewell *et al.* data, upon which spektr 2.0 is based, sums both the *Kα*1 (57.98 keV) and *Kα*2 (59.32 keV) tungsten edges into the 60 keV bin;^55^ spektr 3.0, on the other hand, better resolves the characteristic radiation in 1 keV bins.^20^ Similarly for the high-energy bins, while spektr 2.0 likely suffers from inaccurate polynomial fitting in the 135 keV energy bin,^54^ spektr 3.0 uses a spectral model defined up to 640 kV (Ref. 20) and produces a more accurate fit. Although the discrepancy in the high-energy bin appears large in terms of percent difference, the error is small in terms of the absolute fluence.

## 3. spektrSpectrum() PARAMETRIZATION

### 3.A. Matching spektr 2.0 (TASMIP) and spektr 3.0 (TASMICS)

Due to differences in model generation parameters (e.g., inherent filtration and normalization), TASMICS/spektr 3.0 and TASMIP/spektr 2.0 differ in their basic spectra and tube output characteristics, e.g., the mGy/mAs at 100 cm from the source. To account for such differences, spektr 3.0 includes optional inherent filtration by 1.6 mm Al to match the inherent filtration of TASMIP. Normalizing the tube output (mGy/mAs at 100 cm from the source) of spektr 3.0 calculations to those of spektr 2.0 provides a close match between spektr 2.0 and 3.0 spectra for beam energies 30–140 kV as shown in Fig. 1(b). The ratio of the tube output values for spektr 2.0 and a 1.6 mm Al inherently filtered spektr 3.0 was stored in *spektrScaleFactors.m* for use by other functions in the spektr library. The option to inherently filter with 1.6 mm Al and scale a spektr 3.0 spectra is exercised via the normalize flag in Eq. (1) and is implemented as default (normalize = 1) in *spektrSpectrum*() to provide backward consistency. We matched spectra in terms of mGy/mAs as a simple, convenient method to check and compare calculated vs measured tube output. The spectra could alternatively be matched in terms of HVL or other beam quality characteristics and modifying *spektrScaleFactors.m* accordingly to provide a best match.

### 3.B. Optimization (“tuning”) of spektr input parameters

**FIG. 2.**
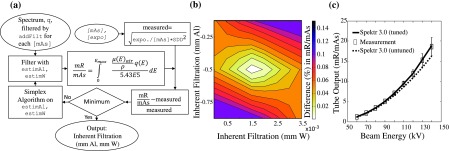
Tuning of spektr calculations for a particular x-ray tube. (a) Flowchart for the *spektrTuner*() optimization using in-air exposure measurements. (b) Illustration of the 2D search space over Al and W thickness (with a normalized 60 kV TASMICS spectrum) to match measured tube output (mR/mAs). (c) Validation of spektr tuning at various kV using a normalized TASMICS spectrum.

An optimization tool called *spektrTuner*() was developed to assist in matching spectral calculations to measurements for a particular x-ray tube in terms of the output (mGy/mAs or mR/mAs). Similar to the process used by Sisniega, Desco, and Vaquero,^23^ the algorithm performs an optimization to match calculated and measured outputs through variation of the thickness of Al and W filtration assumed in the calculation. Larger anode angles typically require higher filtration thickness due to the larger effective path of x-rays produced at depth within the anode. The optimization algorithm and an illustration of the 2D search space are illustrated in Fig. 2 for in-air exposure measurements.

There are several means by which spektr calculations could be tuned to match the output of a particular x-ray tube, e.g., adjusting added filtration to match the HVL and/or mGy/mAs measured at various kV. The *spektrTuner*() function performs a best-fit between measured and calculated tube output as follows. The user first measures either in-air exposure (mR) or air kerma (mGy) at a particular kV and source-detector distance (SDD) at *M* settings of mAs, storing measurements in a [*M* × 1] vector called measurements and the mAs settings in a [*M* × 1] vector called mAs. The *spektrTuner*() function is called with the following arguments:

spektrTuner([spectrum],[mAs],[measurements],SDD,[addFilt],measureFlag,[estimAl;estimW]) (2)

As illustrated in Fig. 2(a), the function first approximates the measured beam via spectrum, a [150 × 1] vector with units of photons/mm^2^/mAs at 100 cm from the source generated using *spektrSpectrum*() at the specified measurement kV. Added filtration in the measured beam is accounted for via addFilt, where each row contains a filter element (atomic number) and its corresponding thickness (mm). The density of the filter material is assumed to be that reported by NIST for the element at standard temperature and pressure and can be retrieved via the function *spektrMuRhoElement*(). Measurements are normalized by mAs and scaled to SDD = 100 cm by the inverse-square law. The user provides an estimate of the inherent filtration, estimAl and estimW, as a [2 × 2] vector representing two material types [viz., Al (*Z* = 13) and W (*Z* = 74) in the first column and their respective thicknesses in the second (e.g., 2 and 0.01 mm)]. In the presence of multiple minima, the minimum closest to the estimates is returned. The tube output (mGy/mAs or mR/mAs) is computed and compared to the quotient of the measurements and mAs arguments. The sum-of-squared-difference is taken as a cost function that is minimized by adjusting the inherent Al and W filtration estimation via the simplex algorithm [computed using the matlab *fminsearch*() function]. Figure 2(b) shows an illustrative sweep across the search space of Al thickness (0.2–0.75 mm) and W thickness (0–0.0035 mm) and the optimal combination that minimizes the objective function. The *spektrTuner*() function returns a [2 × 2] vector containing the atomic number and thickness (mm) of Al and W that provides a best match to the measured tube output.

The measurements in Fig. 2 were obtained on an x-ray imaging bench^59^ incorporating an x-ray tube (RAD13, 16° anode angle, and 0.4 mm focal spot size, Varian, Salt Lake City, UT) and silicon diode (Diagnostic Dose Diode, RadCal Corporation, Monrovia, CA) placed in air at SDD = 745 mm. Measurements were collected over the range 60–140 kV at six mAs stations each. A separate tuning of Al and W filtration was computed for each kV, showing close agreement between measurement and calculation (Pearson coefficient *R*^2^ = 0.98) as shown in Fig. 2(c).

## 4. OTHER ENHANCEMENTS TO spektr FUNCTIONS AND UI

**FIG. 3.**
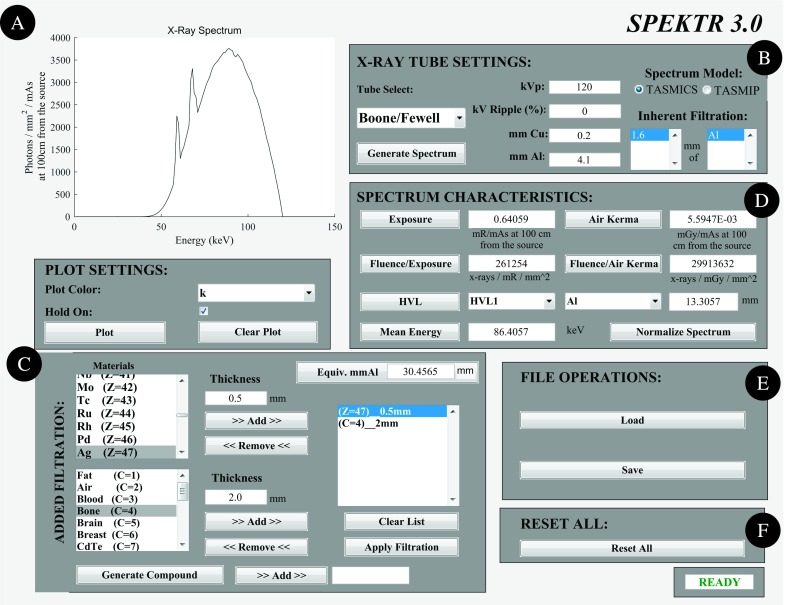
The UI accompanying the spektr 3.0 function library. The UI allows users to generate x-ray spectra, modify filtration, and calculate beam-quality characteristics. (A) Plotting. (B) X-ray tube settings. (C) Added filtration. (D) Spectrum characteristics. (E) File operations. (F) Reset all.

Aside from the underlying TASMICS parameterization, a variety of enhancements and bug fixes have been implemented in spektr 3.0. For example, the UI window (shown in Fig. 3) permits automatic resizing, the Spatial Filter interface has been removed, and all Excel file dependencies from the original release were eliminated. As illustrated in Fig. 3(A), the plotting tool includes standard tools for automatic axis scaling, pan, zoom, and a data cursor.

As illustrated in Fig. 3(B), the underlying spectral parameterization can be selected via radio button between TASMICS and TASMIP. The “Tube Select” drop-down menu has also been modified to automatically display the inherent filtration for any of the tube presets stored therein. The parameters for each tube can be adjusted within a new function, *tubeSettings*(), which keeps a “library” of tube presets with various filtration and kV ripple settings. A field was also added to the x-ray tube settings frame to allow addition of Cu filtration in the basic spectrum calculation.

The Added Filtration tool, shown in Fig. 3(C), was improved by modifying the underlying function, *spektrBeers*(), to accept either the element atomic number (*Z*) or chemical symbol (as a string). Similarly for filtration by compounds, the function *spektrBeersCompoundsNIST*() was modified to accept either the compound index (*C*) listed in *spektrCompoundList.m* or the compound name (as a string). Also, a bug was corrected involving an error in the density of GaAs and Gd_2_O_2_S (5.31 and 7.44 gm/cm^3^, respectively) which were reversed in the original spektr release.

As shown in Fig. 3(D), spektr 3.0 calculates a variety of basic metrics associated with a given spectrum, including exposure (mR/mAs at 100 cm from the focal spot), air kerma (mGy/mAs at 100 cm from the focal spot), HVL (as well as 2nd HVL, 3rd HVL for any element), conversion of the absolute spectrum (photons/mm^2^/mAs at 100 cm from the source) to a normalized probability density spectrum, fluence per unit exposure (x-rays/mm^2^/mR), fluence per unit air kerma (x-rays/mm^2^/mGy), and mean energy (keV).

Finally, as shown in Fig. 3(E), the ability to load previously computed spectra was updated with a simple Load button with folder browsing, and similarly for saving a spectrum via a simple Save button. A pushbutton was created to reset all fields in spektr to default values and clear variables from memory.

## 5. SUMMARY

With the development of the TASMICS algorithm^56^ offering higher spectral resolution, broader energy range, and improved overall spectral characteristics with respect to modern x-ray tubes, this work presents an updated implementation of the spektr function library and UI for research in medical physics and x-ray imaging. A key improvement in this model is to avoid the errors associated with energy bin interpolation in the previous TASMIP and spektr tools.

Despite this improvement, slight differences can be expected between spektr 3.0 calculations and measurements of x-ray spectra or tube output characteristics due to differences in anode angle. To help mitigate such differences, the spektr code scales fluence calculations for beam energies between 20 and 150 kV with the option to match the tube output (mGy/mAs at 100 cm from the x-ray source) computed by TASMICS to that in the previous spektr 2.0 implementation (which in turn matches the measurements by Fewell *et al.*^55^). The resulting spektr 3.0 calculations match within a ∼4% error in mGy/mAs over the range 10–150 keV. This value is similar to the mean percent difference calculated by Hernandez and Boone (2.7%).^20^

Moreover, spektr 3.0 includes a new utility to help users match spectral calculations to measurements with a particular x-ray tube using the *spektrTuner*() optimization. This utility computes the thickness of Al and W filtration (positive or negative thickness) that minimizes the sum-of-squared difference between measured and calculated tube output (mGy/mAs or mR/mAs) using a simplex optimization. Measurements on an x-ray imaging bench demonstrated agreement with tuned spektr calculations with a Pearson Coefficient *R*^2^ = 0.98 for beam energies ranging 60–140 kV. Alternative forms of *spektrTuner*() could be developed to perform best match to other objective functions, for example, HVL. Other modifications improved the functionality of the UI, including better display of inherent filtration for various x-ray tube presets, removal of Excel dependencies, simplification of the input parameters in the *spektrBeers*() and *spektrBeersCompoundNIST*(), and automatic resizing of the UI.

These enhancements update spektr to provide a matlab interface to the TASMICS x-ray spectrum parameterization and will hopefully be of use to researchers in x-ray spectral analysis, image quality modeling, MC simulations, polyenergetic image reconstruction algorithms, and other areas of research for systems in the diagnostic x-ray energy range.
